# Challenging diagnoses of tetraploidy/diploidy and trisomy 12: utility of first-tier prenatal testing methods

**DOI:** 10.3389/fgene.2023.1258752

**Published:** 2023-11-15

**Authors:** Irina Ioana Iordanescu, Anca Teodora Neacsu, Andreea Catana, Zina Barabas-CuzmicI, Viorel Suciu, Cristina Dragomir, Diana Elena Voicu, Emilia Severin, Mariela Sanda Militaru

**Affiliations:** ^1^ Genetics Department “Carol Davila” University of Medicine and Pharmacy, Bucharest, Romania; ^2^ Genetics Department Laboratory, Regina Maria Hospital, Romania; ^3^ Genetics Departament, University of Medicine and Pharmacy “Iuliu Hatieganu”, Cluj-Napoca, Romania; ^4^ Pelican Hospital, Oradea, Romania

**Keywords:** tetraploidy, mosaicism, karyotype, SNP array, abnormal ultrasound

## Abstract

**Introduction:** Chromosome mosaicism and low-grade mosaicism present a challenge for diagnosis in the era of SNP array and NGS. Tetraploidy is a rare numerical chromosomal abnormality characterized by the presence of four copies of each chromosome. The prevalence of tetraploidy/diploidy mosaicism cases is extremely rare in the human population. Accurate estimates of the frequency of this chromosomal anomaly are lacking due to its classification as an extremely rare and difficult-to-detect condition.

**Methods:** In this report, we describe two cases involving challenging diagnoses of tetraploidy/diploidy and trisomy 12. We utilized advanced genetic testing techniques, including SNP array, to examine the chromosomal abnormalities in these cases. We compared the results from SNP array to conventional G band karyotyping to assess the utility of first-tier prenatal testing methods.

**Results:**Our analysis revealed two cases of tetraploidy/diploidy and trisomy 12 with atypical presentations. SNP array analysis provided higher resolution and more precise information about the chromosomal anomalies in these cases compared to conventional G band karyotyping. Additionally, the prevalence of tetraploidy/diploidy mosaicism was confirmed to be extremely rare in the population.

**Discussion:** Low-level mosaicism is difficult to diagnose, and in many cases, it has traditionally been identified through techniques such as G band karyotype or FISH. Microarray has become an invaluable diagnostic tool for detecting chromosomal abnormalities, offering high-resolution insights. However, it may not always be able to detect rare occurrences of tetraploidy or tetraploidy/diploidy mosaicism. As a result, it is recommended to perform a G band karyotype analysis after obtaining a negative microarray result before considering other diagnostic methods with a potentially higher yield of diagnosis. For the detection of low-level mosaicism, combined diagnostic methods should be considered. The diagnosis of mosaicism is a multistep process that can be time-consuming, often requiring the application of more than one diagnostic technique. This approach is crucial for accurate diagnosis and comprehensive patient care. Further research is warranted to better understand the underlying mechanisms of these rare chromosomal anomalies and to develop more effective diagnostic strategies for challenging cases.

## Introduction

The diagnosis of chromosomal mosaicism in the prenatal stage is received with uncertainty and multiple factors need to be considered in order to ascertain the likely impact. The clinical effects of chromosomal mosaicism depend on the type of the imbalance, the timing of the initial event leading to mosaicism and the distribution of the abnormal cells throughout the various tissues within the body as well as the ratio of normal/abnormal cells within each of those tissues. Genetic counseling is an important part for the patients to learn about the possibility of having a child with a chromosome abnormality and these risks differ according to the time of ascertainment and the tissue where the mosaic cells were initially discovered. (Levy et al., 2021).

Tetraploidy is a rare chromosome abnormality characterized by the presence of four copies of each chromosome. It is very rare and in most cases a pregnancy with tetraploidy results in early miscarriage or if the pregnancy goes full term the life expectancy is very short (Bothur-Nowacka et al., 2013). The phenotype varies and is very severe with intrauterine growth restriction, multiple congenital abnormalities, developmental delay (Tetraploidy syndrome, 2023).

Tetraploidy/diploidy is an extremely uncommon condition in humans and most surviving patients are mosaic (Schacht et al., 2017; Tetraploidy syndrome, 2023). In cases of tetraploidy/diploidy mosaicism, there are two populations of cells with distinct karyotypes, with one having 92 chromosomes (tetraploidy) and the other having 46 chromosomes (normal diploidy).

Trisomy 12 is a rare aneuploidy and fetuses with this defect tend to spontaneously abort. However, chromosome 12 mosaicism can result in live births. Mosaic trisomy 12 is encountered more frequently than expected at a rate of 1 in 500 live births (Roberts et al., 2016). Mosaic trisomy 12 at amniocentesis is one of the more frequently described mosaicisms, and often implies a high risk (Gardner and Amor, 2018).

The combination of mosaicism leads to variable phenotypes ranging from normal phenotype to Potter sequence to gross physical defects of the various organ systems (Roberts et al., 2016).

## Case report 1

The first case presented in this study involves a 43-year-old woman in her fourth pregnancy with no reported consanguinity or family history of genetic disorders.

### Previous pregnancies

The patient had experienced two previous pregnancy losses at 27 and 30 years old, respectively, at 8 weeks of gestation. However, she successfully gave birth to a healthy child via C-section at 34 years old, although the child had intrauterine growth restriction.

### Current pregnancy

**FIGURE 1:**
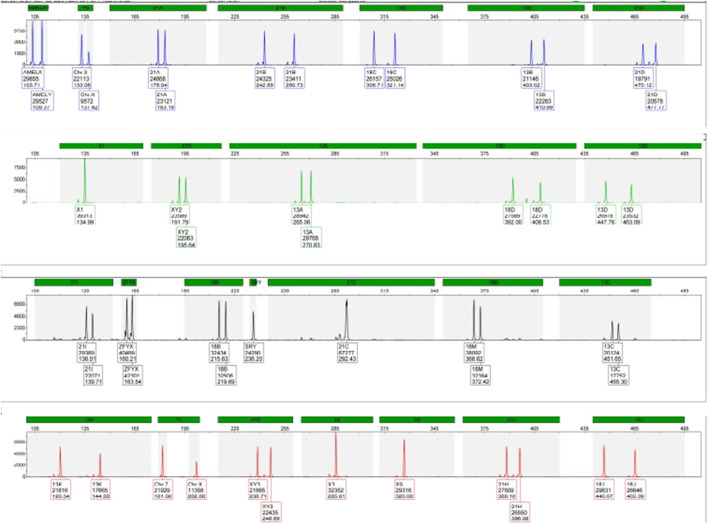
QF-PCR: no aneuploidies were detected for chomosomes 13,18,21, X, Y.

**FIGURE 2:**
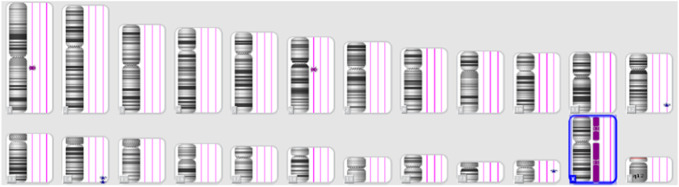
SNP array: arr (X, Y)1 (1–22)x2.

During the current fourth pregnancy, the patient was found to be at high risk for trisomy 21 during the double test at a ratio of 1:25. As a result, QF-PCT and SNP array testing were performed at 16 weeks of gestation, which revealed a normal male foetus, as shown in Figures 1, 2.

### Prenatal diagnosis

**FIGURE 3:**
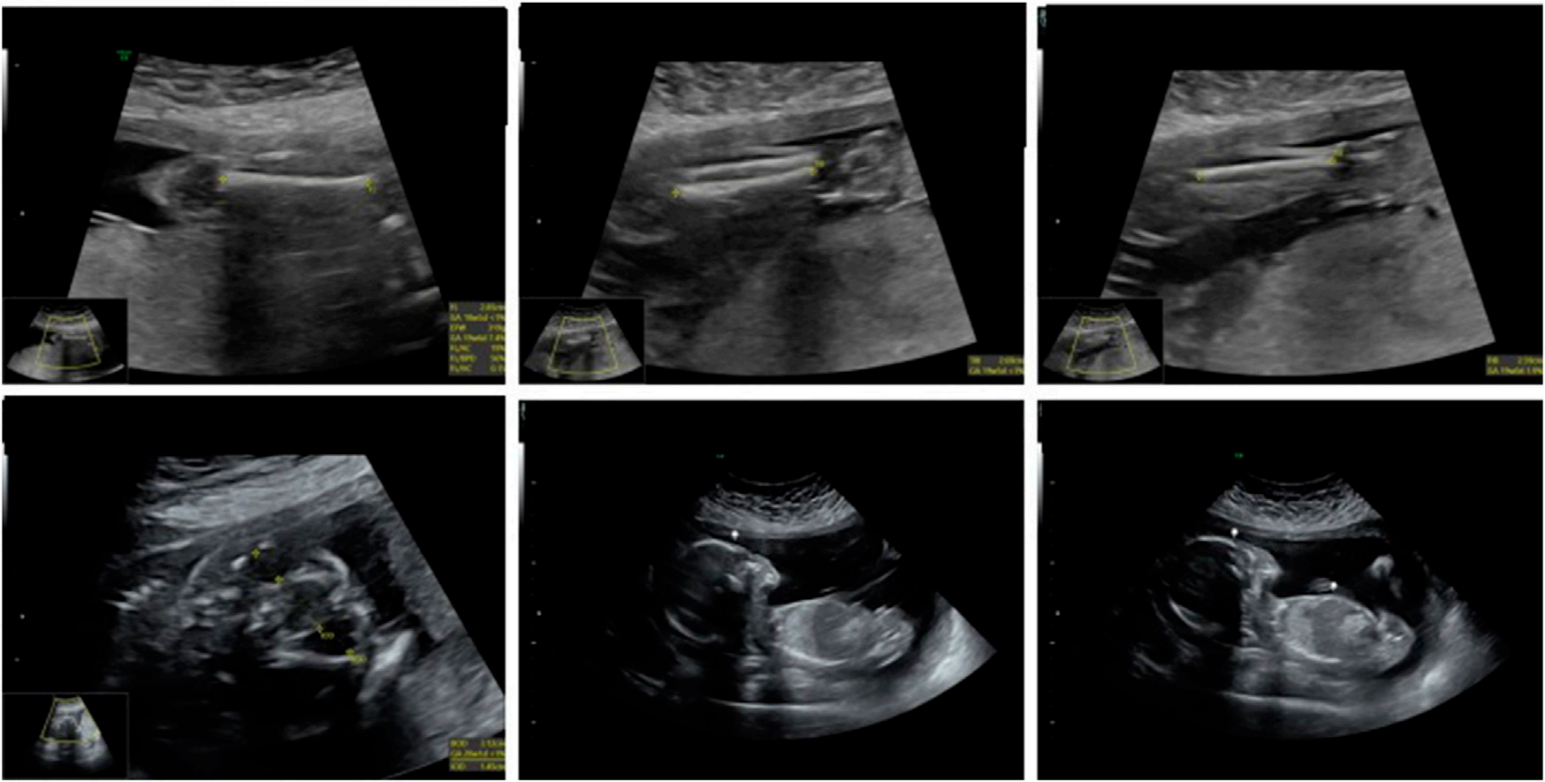
Ultrasound images at 22 weeks of gestation.

A 21-week ultrasound was performed and amniotic fluid was preserved in case of intrauterine death, followed by testing for cystic fibrosis, achondroplasia, and CMV. A subsequent ultrasound at 22 weeks and 4 days showed signs of severe intrauterine growth restriction, with the placenta thicker than at the previous examination and several other abnormalities. (Figure 3).

**FIGURE 4:**
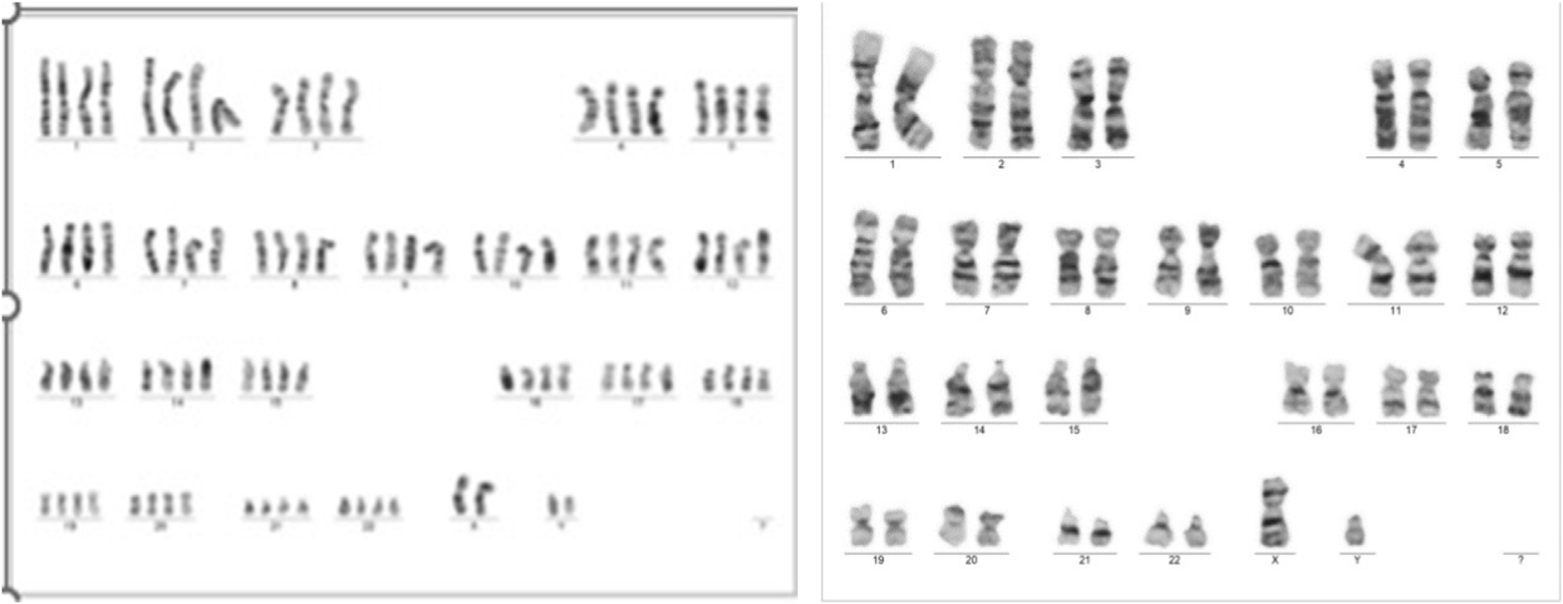
Case 1 G-band Karyotype: 92, XXYY [26]/46, XY (Hay et al., 2018)

Despite these concerns, all test results came back negative. A G-band fetal karyotype was then requested and revealed a mosaic tetraploidy with two karyotypically distinct populations of cells. Specifically, there were 26 cells with 92 chromosomes (XXYY tetraploidy) and 8 cells with the normal diploid karyotype of 46 chromosomes (XY). This is shown in Figure 4. So, this condition can cause severe developmental abnormalities and has a poor prognosis.

## Case report 2

**FIGURE 5:**
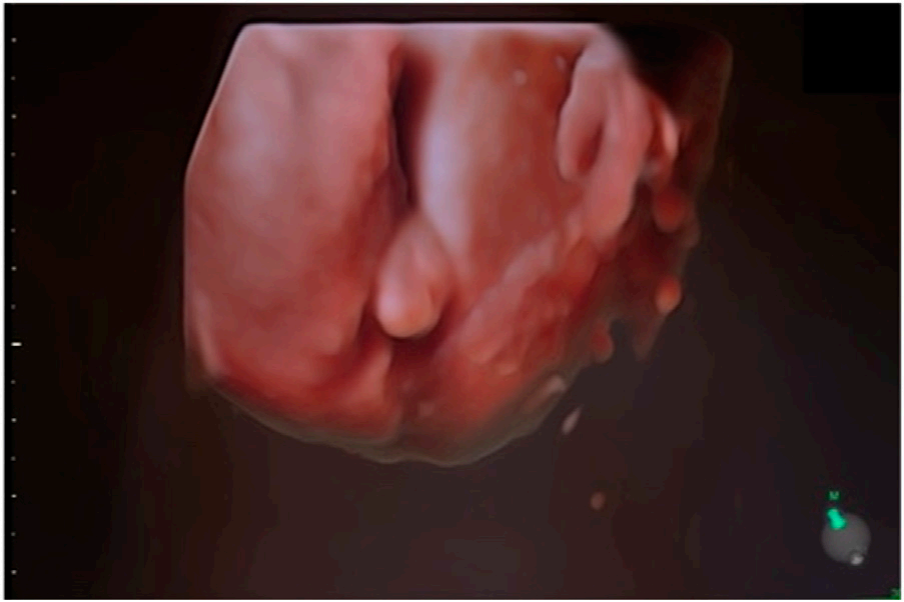
Ultrasonic view of the male fetal external genitalia (20 weeks of gestation, 3D static ultrasound examination).

The second case in this study involves a 31-year-old woman in her first pregnancy. She underwent noninvasive prenatal testing, which indicated low risk for trisomy 13, 18, 21, and male fetal sex. The initial three ultrasounds showed a fetus with female genitalia. However, during the fourth consultation at 20 weeks, the genitalia started to exhibit more male characteristics. (Figure 5). Subsequently, QF-PCR, SNP array, and a panel for sex development disorders were conducted.

**FIGURE 6:**
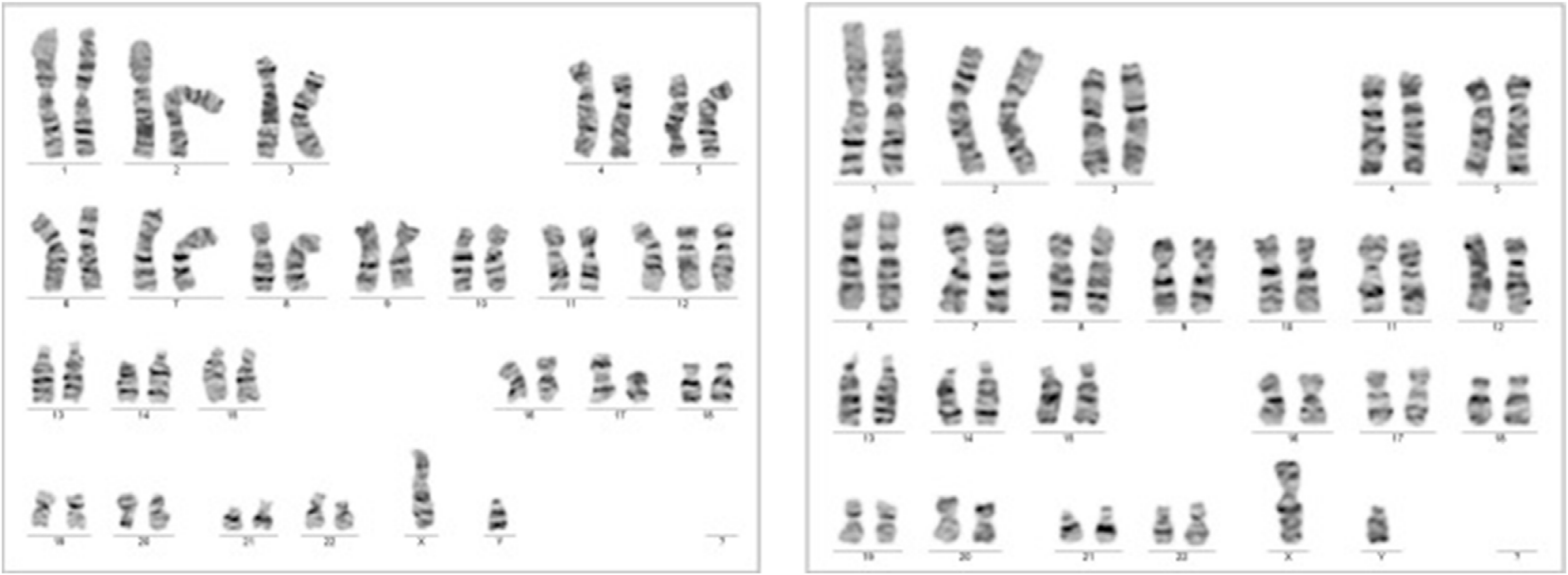
Case 2 G-Band karyotype: 47, XY, +12 (Roberts et al., 2016)/46,XY [25].

The results of the QF-PCR indicated a normal male fetus. The SNP array analysis revealed that the male fetus had a loss of heterozygosity on chromosome 12. The NGS panel conducted did not show any abnormalities, but there were differences in the coverage of five genes on chromosome 12, suggesting a potential low-grade mosaicism for trisomy 12. Following these findings, a new amniocentesis was conducted, and a G band karyotype analysis was performed. The results of the karyotype analysis are shown in Figure 6.

**FIGURE 7:**
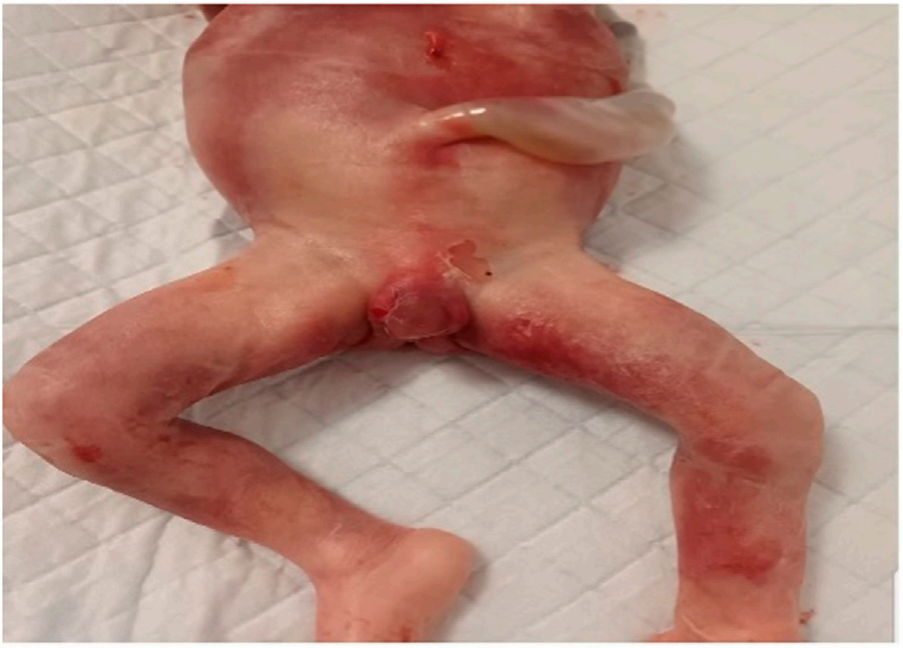
Abnormalities in the appearance of fetal male external genitalia.

Due to the uncertainty surrounding the phenotypic severity and the unknown cause of the sex abnormality, the patient made the decision to terminate the pregnancy. The fetus had severe genitalia malformations. The histopathological examination of the fetus revealed the presence of testes bilaterally in the intraabdominal region. The seminiferous tubules appeared solid with no defined lumen and were filled with Sertoli cells, while Leydig cells were present in the interstitium. The epididymis displayed well-defined tubular structures with a high epithelium and ciliary pseudostratification. Please refer to Figure 7 for further details.

## Materials and methods

QF-PCR was performed using the Devyser v3 compact kit for amplification, detection, and analysis of chromosome-specific DNA sequences known as genetic markers or STRs. Twenty-six genetic markers were used, labeled with fluorescent probes, and were analyzed and quantified using an automated genetic analyzer (ABI 3500, Applied Biosystems). The interpretation was performed using the GeneMapper software (Applied Biosystems).

The hybridization method was performed using the Affymetrix CytoScan 750 K platform. The resolution is 100 kb, having a hybrid design that allows the CNV detection, can determine the exact breaking points, identify LOH, triploidy, having 750.000 markers for copy number and 200,000 genotype-able SNPs which provide high resolution copy number, accurate breakpoint estimation, and loss of heterozygosity (LOH) detection. The databases used are Chromosome Analysis Suite (ChAS) Software, NCBI Build GRCh38 (hg38), ClinVar, OMIM (Online Mendelian Inheritance in Man), NCBI, DECIPHER. This technique does not detect balanced translocations, inversions, point mutations and low-grade mosaicism. The limit for SNP array for mosaicism is 15%–20%.

Karyotype was performed on amniocyte culture. After G banding the slides were analyzed using the Nikon automated microscope, and the Lucia Karyo software. For each sample 20 metaphases are karyotyped, and if there is one metaphase with a different cell line additional metaphases are analyzed.

Written informed consent was obtained from the two pregnant women for the publication of any potentially identifiable images or data included in this article.

## Discussion

Tetraploidy is a condition characterized by the presence of four complete sets of chromosomes in a single cell. It is typically caused by the duplication of chromosomes in a somatic cell during the early cleavage stage of embryo development, occurring after fertilization (Levy et al., 2021). Tetraploidy is relatively common in cases of pregnancy loss, accounting for approximately 5% of first-trimester losses. In rare instances where the pregnancy progresses and the infant is born, most cases exhibit mosaicism, meaning that the cells contain a mixture of tetraploid and diploid chromosomes. Tetraploid/diploid mosaicism has been reported in only 16 patients, displaying a diverse range of clinical presentations (Schacht et al., 2017).

In recent years, chromosomal microarray analysis (CMA) has gained prominence in prenatal diagnosis. This method has significantly improved the detection rate of clinically significant chromosomal abnormalities. By utilizing high-resolution single nucleotide polymorphism (SNP)-based microarrays, CMA can identify microdeletions, microduplications smaller than 5–10 Mb (with higher resolution than traditional karyotyping), whole chromosome aneuploidies, unbalanced rearrangements, triploidy, uniparental isodisomy, and low-level mosaicism. Karyotyping, on the other hand, is limited to the detection of whole chromosome aneuploidies, large deletions, and duplications (≥5–10 Mb), polyploidy, and some balanced chromosomal rearrangements (Cavalcanti and Zanchetta, 2005).

A multicenter study on microarray analysis conducted by the National Institute of Child Health and Human Development (NICHD) in 2012 demonstrated 100% agreement between microarray results and traditional karyotyping for prenatal diagnosis of aneuploidies (Hay et al., 2018). Following the publication of this study, the American Collage of Obstetricians and Gynecologists (ACOG) and the Society of Maternal-Fetal Medicine (SMFM) recommended microarray analysis for pregnancies with ultrasound findings, and for pregnancies without ultrasound findings, either microarray or karyotype could be considered (Levy and Wapner, 2018). Many physicians now advise microarray analysis for pregnancies with multiple ultrasound findings. The SNP array, a more advanced and reliable method for detecting chromosomal abnormalities, offers improved resolution and has a diagnostic yield of 8% higher than karyotyping. Consequently, it is widely employed as the initial diagnostic test in prenatal settings (Kamath et al., 2022).

Trisomy 12 in mosaicism is a rare condition, and the literature reports only a few cases of live births. The clinical presentation of trisomy 12 mosaicism is highly variable, ranging from lethality in the newborn period to features such as global developmental delay, congenital heart disease, microcephaly, cutaneous spots, dysmorphism, hypotonia, retinopathy, and sensorineural hearing loss. Two other cases of trisomy 12 mosaicism have been reported: an adult investigated for infertility, while the other exhibited overgrowth (DeLozier-Blanchet et al., 2000; Gasparini et al., 2019). Additionally, there have been reports of individuals with trisomy 12 mosaicism who do not display any phenotypic abnormalities. Consequently, prenatal genetic counseling for cases involving trisomy 12 mosaicism is challenging due to the lack of known correlations between the extent of mosaicism and the clinical outcome (Hainz et al., 2021).

A study from 2023 reported 44 cases of mosaicism detected using SNP array. Most cases were from chorionic villus sampling (but no exclusion of confined placental mosaicism was performed). In this study there were also deletion/duplications mosaicism. The concordance with karyotype was 66,7%, and the percentage of the mosaicism was greater than 20% (Zhou et al., 2023).

In another study published in 2020 on a cohort of 5,000 pregnancies, it is revealed that from 19 cases of mosaic detected by karyotype 6 were not detected by SNP array, 3 due to low level aneuploidy mosaicism and 3 due to structural rearrangements (Xiang et al., 2020).

As diagnostic methods continue to evolve, techniques such as whole exome sequencing (WES) and even whole genome sequencing (WGS), whether conducted individually or in trio, are increasingly employed in prenatal settings. However, these methods have a few limitations when it comes to detecting conditions like tetraploidy or low-grade mosaicism.

To the best of our knowledge from the literature, there is only one reported case of a normal man with Kartagener syndrome and true 12 mosaicism investigated for infertility (DeLozier-Blanchet et al., 2000). Our case presented development abnormalities such as ambiguous genitalia and malformation. No other abnormalities were identified during the examination.

Prenatal genetic counseling for cases involving tetraploidy or low-grade mosaicism can be challenging due to the lack of known correlations between the extent of mosaicism and the clinical outcome. The clinical phenotype associated with trisomy 12 in mosaicism is highly variable and can encompass a range of developmental delays, congenital heart disease, microcephaly, cutaneous spots, dysmorphism, hypotonia, retinopathy, sensorineural hearing loss, and, in rare cases, overgrowth. However, there have also been reports of individuals with trisomy 12 mosaicism showing no phenotypic abnormalities.

In cases involving tetraploidy or low-grade mosaicism, several recommendations can be made:

### Prenatal genetic counseling

Genetic counseling is crucial to provide information and support to individuals or couples facing these conditions. A genetic counselor can explain the nature of tetraploidy or mosaicism, discuss the associated risks and uncertainties, and help individuals make informed decisions about further diagnostic tests or pregnancy management options.

A clear diagnosis is an essential base for accurate genetic counseling. The genetic counselor must give the patient all the information regarding the evolution of the pregnancy and management for future pregnancies in a nondirective approach and to help the individuals make their own decision (Clarke, 2020).

### Comprehensive prenatal testing

Given the limitations of traditional karyotyping in detecting tetraploidy or microdeletions/microduplications, the use of chromosomal microarray analysis (CMA) is recommended. CMA offers higher resolution and can detect smaller chromosomal abnormalities, including microdeletions and microduplications. Incorporating CMA as part of routine prenatal testing can provide more comprehensive information about the genetic status of the fetus.

### Consideration of advanced sequencing techniques

In certain cases, when available and appropriate, advanced techniques such as whole exome sequencing (WES) or whole genome sequencing (WGS) may be considered. These methods can provide a more detailed analysis of the entire genetic code, potentially identifying specific genetic variants or mutations that may be responsible for the observed abnormalities.

### Multidisciplinary care

Due to the variable clinical presentation and potential associated health issues, a multidisciplinary approach to care is beneficial. Involving specialists from various fields such as genetics, pediatrics, neonatology, cardiology, and developmental services can ensure comprehensive management and appropriate interventions tailored to the specific needs of the individual.

### Long-term follow-up

Individuals with tetraploidy or mosaic trisomies may require long-term follow-up and monitoring for potential developmental delays, congenital anomalies, or other associated health conditions. Regular assessments, including developmental screenings, cardiac evaluations, and other relevant tests, should be conducted to address any emerging health concerns promptly.

It is important to note that recommendations may vary depending on the specific circumstances and individual cases. Therefore, personalized medical advice from healthcare professionals and genetic specialists should always be sought to determine the most appropriate course of action.

### Conclusion

Tetraploidy or tetraploidy/diploidy mosaicism is a rare chromosomal abnormality that can be detected by G band karyotyping.

Low level mosaicism is difficult to diagnose. Most cases are diagnosed with G band karyotype or FISH.

Microarray is another diagnostic tool that can be used to detect chromosomal abnormalities, but it may not be able to detect tetraploidy or tetraploidy/diploidy mosaicism in some cases. Therefore, it is recommended to perform a G band karyotype after a negative microarray result before considering other diagnostic methods with better yield of diagnosis. For the detection of low-level mosaicism combined methods of diagnosis should be performed. The diagnosis of mosaicism is a multistep process and time consuming often more than one diagnostic technique being required.

## Data Availability

The raw data supporting the conclusion of this article will be made available by the authors, without undue reservation.
